# Dental Health Knowledge Attitude and Practice Among University of Calabar Students

**DOI:** 10.7759/cureus.40055

**Published:** 2023-06-06

**Authors:** Caroline C Okoroafor, Okelue E Okobi, Moravia Owodeha-ashaka, Emeka Okobi, Babadamilola Oluseye, Oritsegbemi B Ekpang, Lucky E Aya, Oluwasayo J Owolabi, Tiku-etah Oru-betem, Jane N Nwafor

**Affiliations:** 1 General Practice, University of Calabar, Calabar, NGA; 2 Family Medicine, Arizona State University, Tempe, USA; 3 Family Medicine, Lakeside Medical Center, Belle Glade, USA; 4 General Practice, University of Calabar Teaching Hospital, Calabar, NGA; 5 Dentistry, Ahmadu Bello University Teaching Hospital Zaria, Abuja, NGA; 6 Oral Surgery, Lagos University Teaching Hospital, Lagos, NGA; 7 Hematology, University of Calabar Teaching Hospital, Calabar, NGA; 8 Family Medicine, University of Calabar Teaching Hospital, Calabar, NGA; 9 Psychiatry, Lugansk State Medical University, Lugansk, UKR; 10 General Medicine, University of Calabar Teaching Hospital, Calabar, NGA; 11 Internal Medicine, University of the District of Columbia, Silverspring, USA

**Keywords:** dental practice, attitude to dental care, dental care, students, oral health, dental health

## Abstract

Introduction

Oral health has been linked to aspects of individual knowledge, attitude, and practices. In Nigeria, the increased prevalence of poor oral hygiene has been attributed behavioral factors. Behavioral aspects that include the increased intake of sugary foods and beverages, and lack of proper oral hygiene have been touted as the main causes of poor oral hygiene among university students. The knowledge of oral health is vital owing to its contribution to better oral health; however, unless students develop good oral habits and attitudes and subsequently put them into practice, very little will be realized with regard improvement in oral health and hygiene.

Objective

This research aimed to explore the knowledge, attitude, and practice of dental care among the University of Calabar students.

Method

This study was a descriptive cross-sectional study conducted between 2016 and 2017. A standardized questionnaire was used to collect data from 430 student participants in a university using a multi-stage random technique. An inferential statistical approach was adopted to test the relationships represented in the tables. Data were analyzed using the statistical package for social sciences, version 20.0.

Result

The study included 430 participants, 239 (55.6%) females and 191 (44.4%) males. The survey revealed that 94% of the 404 respondents agreed that poor dental care can cause dental diseases, while only 6% disagreed. Regarding excessive drinking of water, 91% of the respondents agreed that it cannot cause dental disease, 4.2% agreed it could, and 4.8% did not know. Furthermore, 60.2% of the 430 respondents acknowledged that genetic inheritance could cause dental disease, while 21.4% disagreed, and 18.4% did not know. Finally, 74.9% of the respondents knew that trauma to the teeth could cause dental disease, while only 9.3% thought that trauma could not cause illness to the teeth.

Regarding attitude to dental care, 232 (54%) respondents agreed that visiting the dentist was necessary, while 164 (38.1%) strongly agreed. Only eight (1.9%) strongly disagreed, while five (1.2%) disagreed that visiting the dentist was necessary. Moreover, 82% of the respondents agreed that bad breath was associated with poor dental care, with 195 (45.3%) respondents strongly agreeing and 158 (36.7%) agreeing. However, 37 (8.6%) disagreed, and 16 (3.7%) strongly disagreed, while 24 (5.6%) were indifferent.

As regards practice, most respondents used the up-down technique when brushing their teeth (62.8%), while 17.4% brushed left-right and 19.8% used both methods. Moreover, 67.4% of the respondents brushed twice daily, 26.5% brushed once daily, and only 6.1% brushed after every meal. About half of the students spent one to three minutes brushing their teeth (50.5%), while the other half spent more time. Over half of the students replaced their toothbrushes every three months (57.7%), with the most common reason for replacement being the fraying of bristles. However, the use of dental floss was found to be low.

Conclusion

The utilization of dental care facilities was low among most University of Calabar students, who did not see the need for dental clinic visits unless they had dental needs. The lack of dental visits was attributed to perceived high dental costs and a lack of time. Targeted interventions and educational programs that address these barriers could promote better oral hygiene practices among the students.

## Introduction

Knowledge of oral health is vital to the maintenance of good oral health: however, poor oral health remains a significant burden for individuals globally and in developing countries [1]. Globally, oral diseases remain key public health concerns worldwide. Population oral health status is determined through evaluation of dental caries and periodontal diseases prevalence, and oral hygiene levels. Startlingly, approximately 60%-90% of school-aged children and nearly all adults globally have dental caries. Additionally, severe periodontal disease affects nearly 15%-20% of adults aged 35 to 45 years. Globally, approximately 30% of people aged 65-74 have lost all their natural teeth [2].

Although data on the global economic costs of oral diseases is unavailable, the World Health Organization estimates that without preventive measures, the treatment of oral diseases might be the fourth most expensive condition to treat. In the US, in 1996, oral diseases led to a loss of 2.4 million working days and 1.6 million school days. Further, in 2008, dental issues resulted in a loss of 1900 school hours per 1,000 children in Thailand, indicating the economic and social implications of oral diseases [3].

Studies conducted in Africa reveal a high prevalence and severity of periodontal disease, which is associated with poor oral hygiene and nutrition [4]. The disease affects all age groups, with a 50% and 75% incidence rate, and increasing, in children and adults, respectively [5]. Data from 39 African countries demonstrate that untreated caries and decayed, missing, or filled teeth (DMFT) among 12-year-olds are very low in 13 countries (33%), low in 19 countries (44%), and moderate in seven countries (23%) [6].

Despite the limited number of oral health studies conducted in Nigeria, periodontal disease has been noted to occur at an early age, with a prevalence of 15%-58% in children aged 15 years and above and is attributable to increased consumption of Western-style diets [7]. Given that oral health is dependent on numerous factors, it is crucial to prioritize oral healthcare beyond just tooth care [8]. While knowledge of the importance of good oral health is essential, it is insufficient for achieving good oral health. Developing and implementing good oral health habits is crucial for improving dental health [9].

Dental caries and periodontal disease are considered behavioral as their treatment and prevention require the adoption of healthy oral habits [10]. Maintaining good oral health requires regular teeth brushing with fluoride toothpaste, flossing, mouthwash use, and bi-annual dental visit [11]. Tooth brushing is the widely practiced method for maintaining good oral hygiene and preventing periodontal disease [12], despite available evidence indicating that tooth brushing only is insufficient in maintaining oral health. Additional methods are recommended. Brushing twice a day and use of dental floss are not common practices in Nigeria. Eighty-seven percent of medical students in Niger Delta University, Bayelsa brushed once daily [13]. Despite being exposed to the dental curriculum, only 47.5% of dental students in Nigeria practice tooth brushing twice daily [14] compared to 81% of their counterparts in Mongolia [15]. Numerous studies have been reported on oral health practices among dental and medical students, and among students in professions allied to medicine. In Nigerian studies, 32% of nursing students [16] and 25% of medical students [13], and 80% of dental auxiliaries in training [17] utilized dental services. The utilization of dental services is often motivated by pain and the need for emergency care.

Statement of problem

Oral disease is linked to individual behavior and poor oral hygiene. Therefore, an improvement in oral hygiene and decreased sugar intake has been shown to reduce the prevalence of dental caries and periodontal diseases [12]. Studies have been carried out on the knowledge, attitude and practice of dental care among dental students in Nigeria [18-24]. However, there is no available data on oral health knowledge and practice among university students who have had no formal exposure to the dental curriculum. Though a weak association exists between oral health knowledge and good dental care practice, knowledge is an important prerequisite for health-related behavioral change [12]. Therefore, there is a need to assess how much the University of Calabar students know about and practice good dental hygiene.

Study objectives

The study objective is to assess the knowledge, attitude, practice, and utilization of dental care services among University of Calabar students. This includes evaluating the level of knowledge of dental care practices, determining the attitude towards dental care practice, assessing the actual practice of dental care, and examining the level of utilization of dental care services by the University of Calabar students.

Knowledge of dental care

Oral health has remained an integral part of an individual’s general health and overall well-being. In order to follow healthy oral habits, it is important to have good knowledge and attitude toward oral health. The knowledge is derived from information and the information, when accepted and believed will be translated into an action which in turn becomes a habit [21]. About 90% of school children worldwide and most adults have experienced caries, with the disease being most prevalent in Asia and Latin American countries. These could be attributed to several factors mainly lack of oral awareness and over consumption of refined carbohydrates [22]. A study carried out in order to identify the extent of dental caries occurrence and to relate it with the dental health knowledge, attitude and practice among adolescents in Ibadan North (LGA) of Oyo State Nigeria found a significantly high knowledge, positive attitude and sound practices towards dental health [23]. Another study carried out among undergraduate non-medical students at the University of Port-Harcourt, Nigeria, showed that approximately 60% of students knew that they had to brush their teeth twice daily, 31% knew they needed to visit the dentist twice a year and only 18% knew what dental floss was [24]. In Ogoja Local Government Area of Cross River State research carried out in a private school showed that most respondents had a fairly good knowledge of basic hygiene measures, a fairly positive attitude towards oral hygiene, and exhibited poor oral hygiene practices in both schools [18].

Attitude towards dental care

Studies carried out in 27 universities in 26 countries across Asia, Africa and the Americas showed that oral health behavior among students was low [20]. Dental care is a crucial aspect of general healthcare. Unfortunately, little or no attention is paid to it. Proper dental care can reduce the presence of bacteria and this will in turn reduce strain on the human immune system. The association between poor dental care and tooth discolorations was extensively studied by Ibiyemi and Taiwo, and Kershaw et al, where most of the respondents stated that tooth discolorations were mainly due to poor oral hygiene [25]. 

In a study carried out among undergraduates at the University of Benin, only about 35.8% of the respondent were aware of tooth bleaching with the main source being the dentist and the internet [26]. A study carried out among preclinical and clinical dental students reveals that a higher percentage of preclinical students worry about the colour of their teeth, and gum, and put off going to the dentist until they have a toothache [27]. According to a study that was conducted in north Jordan [28] on school children averaged 13 years of age (N=557) they found out that oral hygiene habits among them were irregular and the input of their parents was limited. Visits to the dentist were irregular, with toothache being the driving force. Children’s attitude towards dentistry was found to be positive even though they indicated fear towards dental treatment. The children recognized and acknowledged the importance of dental health for the health of their whole body. Parents were not found to practice consistent and regular oral healthcare for their children hence attitude towards dental care, according to the study, should be improved and this is possible only by more health talks and programs for both parents and children. In another study carried out on students in Mafrag Jordan, the results showed that 25% of the people brush twice or more daily, 17% of the students never brushed at all, and 22.1% of rural students never brushed compared to 14% of their urban counterparts. Dental flossing among students in the Mafrag governorate was very rare. Also, 97.2% have never flossed in their life [29]. 

Furthermore, a study was carried out among dental students in India and it was discovered that 70% of dental students agreed with the statement: “I don’t worry much about visiting a dentist”. About 50.7% reported that they postponed their going to the dentist until they had a toothache, 47.2% felt that they took much time to brush their teeth, 78.7% agreed to use mirrors while brushing, 68% were worried about their teeth colour while 46.8% were worried about their gingival color [30]. However, oral health attitudes and behavior, in undeveloped countries especially rural areas are due to a lack of oral healthcare awareness [31]. A study carried out on the determinants of preventive oral health behavior among senior dental students in Nigeria showed that more male respondents agreed that the use of fluoride toothpaste was more important than tooth brushing technique for caries prevention, while the use of dental floss was very low about 7.3%. More females were more likely to comply with recommended oral self-care (7%). Older respondents and younger respondents with more knowledge of preventive dental care were more likely to consume sugary snacks less than once a day.

The frequency of twice-daily brushing among dental students in Nigeria is very low (47.5%) compared to their counterparts in Mongolia (81%), France (78%), Iran (57%) and Australia (80%) and it is even less than what has been reported in the general public in Germany, Japan, USA, New Zealand, UK, Portland, Finland. A survey done in Lagos State University Teaching Hospital (LASUTH) on the knowledge attitude and practice of oral health by pregnant women receiving ANC showed that 62.9% of them were reported to have visited a dental facility. About 37.5% consumed vegetables four times a week, 85% consumed confectionery more than four times a week, 94.2% used toothbrush, 65.1% cleaned their mouth once daily, and 34.2% cleaned their mouth twice or more daily. About 82.8% of adolescents in a secondary school in Oyo strongly agreed that the dentist should be visited once in 6 months while 9.0% disagreed [32]. However, this does not translate to good practice as very few (31.6%) had ever visited the dentist [33].

Practice of dental care

In a comparative study, 44.1% of a group of medical students brushed twice daily and 10.9% after every meal while engineering students' group 39.1% brushed twice daily and only 0.9% brushed after meal. Fraying of the toothbrush was reported to be the most common reason for the renewal of toothbrush among both groups [34]. A study among paramedical students reported that 32% of the sample size agreed to brush once daily, 64.7% brushed twice daily and 3.3% brushed more than twice. About 82.7% used only toothpaste and brush while 17.3% used toothpaste and brush and dental floss [35]. A study among Nursing students reports that the majority of the students brushed twice daily with a toothbrush and toothpaste for a duration of two to three minutes (Laxman et al, 2012). A total of 23.3% of adolescents obtained information on oral health from doctors/dentists, 26% from mass media and 50.7% obtained information from school/college [35].

Dental students showed slightly better self-care results than those of the Turkish non-dental students whose health was assessed. Of the 610 students randomly selected, only 3% used dental floss daily, and 68% of the students brushed their teeth two or more times per day. Thirty per cent visited the dentist for preventive treatment at least once a year [36,37]. A study on dental health knowledge and attitude toward the occurrence of dental caries among adolescents in the Oyo local government area revealed that the practice of dental hygiene among respondents was good. Many of them irrespective of their age groups used good toothbrush and toothpaste, use the up-down and sideways techniques for brushing and claimed to always rinse their mouth with water after each meal. Findings here however showed that only a few of the respondents go for dental check-ups at least once a year [38]. These results are somewhat similar to those found in another cross-sectional survey conducted at the University of Port-Harcourt, Rivers State among non-medical students. Of the 360 respondents 18-33 years, 90% brushed their teeth at least once a day. About 28.8% replaced their toothbrush every three months. Only a few students, 5.8% and 4.2% used dental floss and mouthwash respectively as oral cleaning aids. Most of the students (71.6%) had never visited the dentist, 18.1% visited due to dental pain, and 8.1% for extraction [39]. These results do not seem to support the notion that dental care practice in developing countries is less than the developed countries, as data collected from 27 universities in 26 countries across Asia, Africa and America showed that 67.2% reported brushing their teeth twice or more times in a day, 28.8% about once a day and 4.0% never. 16.3% reported dental check-up visits twice a year, 25.6% once a year, 33.9% rarely and 24.3% never [20].

Comparatively, in a cross-sectional study among adult Danes aged 16 years and above, tooth brushing twice a day was reported by 68% while 32% brushed once a day or less frequently. The use of dental floss was reported by 11%. Here regular dental visits, dental care during school years, and a high level of education were related to tooth brushing twice a day [40]. A study carried out among Dental technology and therapy students in Enugu showed 71.9% of the respondents brushed their teeth twice daily and 52.1% brushed for three to five minutes. About 30.2% brushed their teeth in front of a mirror. Tongue cleaning was done by 94.2% with only 9.5% using tongue cleaner [17].

Utilization of oral healthcare services

A study in India among paramedical students revealed that 56% of students visited the dentist while 44% did not. About 10.7% visited every six months, 14% visited once a year and 75.3% visited when they had pain [35]. Another comparative study in India showed that 68.4% of medical students and 68% of engineering students visited only when they had tooth pain. Only 20% of medical students and 18% the engineering students visited once in 6 months while about 3.2% of medical students and 4.4% of engineering students visited once every month [34]. Another study among Nursing students revealed that the majority visited the dentist only on pain [36] These study shows that the majority of students visited the dentist only when they had tooth pain. In a study of 7, 630 participants in Nigeria, it was found that although overall 21.1% of the participants rated their oral health status as very good, only 26.4% reported having visited the dentist at least once prior to the conduct of the survey. More than half of these visits (54.9%) were for treatment purposes, and the utilization of oral health services was significantly greater than 0.05 than associated with being older, more educated and being engaged in a skilled profession. However, in a study carried out among health workers in Benin City [41] only 18% of the respondents reported visits to the dentist at least once every year. 

In the Jordan study (2014), half of the respondents visited the dentist because of pain or bleeding gums. Females primarily visited the dentist for aesthetic reasons (70%), and males upon complaint (59.8%). A study at the University of Lagos showed that about half (50.9%) of respondents had used dental services in the past 12 months. There was no significant association between age, gender, year of undergraduate education, and faculty of these students and their utilization of oral healthcare services. A high proportion of respondents had attended the dental clinic for dental checkup (33.6%) and extractions (30.9%). The major barrier to receiving dental treatment was a lack of perceived need for treatment (53.1%), followed by lack of time (29.1%), fear (18.2%) and cost of treatment (18.2%).

## Materials and methods

Study setting

Cross River State, in the South-South region of Nigeria, is bordered by Benue State, Ebonyi, Abia, the Republic of Cameroon, Akwa Ibom State, and the Atlantic Ocean. The State comprises 18 local government areas, and its capital is Calabar. The State is home to three major ethnic groups - Efik, Ejagham, and Bekwarra, with the Efiks being the most dominant [42,43]. Calabar has two tertiary institutions, including the University of Calabar, which is a federal institution located in the Calabar South Local Government Area. The university is amongst the largest in Nigeria's South-South geopolitical zone, with about 40,655 students. The university has 12 faculties, including Medicine/Dentistry, Allied Medical Sciences, Basic Medical Sciences, Physical Science, Biological Science, Social Science, Management Science, Education, Law, Agriculture, and Arts. The institution has an annual enrolment of about 5000 students [42,43].

Study design

A descriptive cross-sectional study was conducted over a period of four months from October 2016 to March 2017. The study focused on male and female students at the university, who formed the target population.

Inclusion criteria

All adult male and female students of the University of Calabar who gave consent were included.

Exclusion criteria

The students who had graduated from the institution were excluded from participation.

Sample size determination

The Leslie-kish formula was used to determine the minimum sample size. The formula is written as follows: n=z^2^pq/d^2,^, where n = minimal sample size, Z = standard normal deviation estimated at 1.96 at a 95% confidence interval, P = proportion of desired attribute prevalence, d = acceptable sampling error, the precision of study ±0.05, P = O.5 (Proportion of people with knowledge of Dental Care) [32], q=1-p, q =1 -P = 1-0.5 = 0.5, n = 1.96^2^ x 0.5 x 0.5/0.05^2 ^= 384.16; therefore n = 384, adjusting for non-response, 384xQ, where Q=1/1-f, f= non-response rate = 10% = 0.1, Q = 1/1-0.1 = 1/0.9 = 1.1, 384 x 1.1 = 426.6. Therefore, the estimated sample size = 427.

Sampling technique

Multi-stage sampling technique (in three stages) was used to get 427 study participants. Stage one: Simple random sampling technique by balloting was applied in stage one. Five (5) faculties were selected from 12 faculties in the university. Each faculty was written on a small sheet of paper cut into equal sizes, folded neatly, put into a non-transparent bag, and shuffled. Five papers were picked one at a time from the bag. The faculties picked were Medicine, Basic Medical Sciences, Arts, Social Sciences, and Biological sciences.

In stage two, simple random technique of balloting was applied. Each department was written on a small sheet of paper cut into equal sizes, folded neatly, put into a non-transparent bag, and shuffled. For the faculty of Medicine, medicine and dentistry departments were picked. For the faculty of Basic Medical Sciences, the departments of Anatomy and Biochemistry were picked. For the faculty of Biological Sciences, the departments of Botany and Microbiology were picked. For the faculty of Arts, the departments of Philosophy and English and Literary Studies were picked while for the faculty of Social Sciences, the departments of Political Science and Public Administration were picked. In stage three, participants from each of the picked departments were selected based on convenience.

Method of data collection and data collection tool, and analysis

A semi-structured questionnaire was utilized to gather data through a quantitative research approach. The questionnaire was designed to obtain information from respondents regarding their socio-demographics, knowledge, attitude, practice, and utilization of dental care. Prior to the distribution of the questionnaire, team members briefed the participants on the study and provided instructions on how to complete the questionnaire accurately. The questionnaire was then manually sorted and analyzed using Statistical Package for Social Sciences (version 20.0), with quantitative data summarized using measures of central tendencies and dispersions, such as means, frequencies, and standard deviations. Data were also presented in the form of tables and figures. To test relationships, a regression statistical model and an inferential statistical technique called Chi-Square were employed. The questionnaire was distributed to participants before or after their lectures.

Questionnaire and Ethical Consideration

We developed our questionnaire by adhering to the standard validation process. Initially, we created clear, unambiguous, and unbiased questions, ensuring their psychometric evaluation for validity and reliability, which was done through content validation and construct validation. We carefully assessed their consistency, suitability for the target audience, conciseness, and appropriateness, and ensured mutually exclusive response options. The consistency was determined using test-retest reliability, which was approximated through the calculation of correlations between scores of two of the questionnaires with similar sets of participants. To refine the questionnaire further, we conducted a pilot test with a small sample, enabling us to identify and address any issues or areas for improvement. Based on the evaluation of the pilot test outcomes, we made necessary adjustments and arrived at the final version of the questionnaire. Lastly, our questionnaire obtained approval from the institutional review board. Ethical approval was obtained from the University of Calabar Teaching Hospital Research Ethics Committee (IRB#01212017). Informed consent was obtained from all participants before administering the questionnaire. 

## Results

Socio-demographic characteristics of study participants

A total number of 430 students participated in the study. A total number of 430 questionnaires were administered to students of the University of Calabar. Table 1 shows respondents' percentage and frequency distribution based on their socio-demographic characteristics. The majority of the respondents, 237(55.1%), were 20-23 years, with the least, eight (1.9%), being 33 years and above. There were 101 (23.5%) respondents aged 16-19 years (emancipated minors) and 65 (15.1%) respondents aged 24-27 years. About 19 (4.4%) respondents were aged between 28 and 31 years,

**Table 1:** Social-demographics of participants

VARIABLE	FREQUENCY (%)
Age group/years	
16-19	101 (23.5)
20-23	237 (55.1)
24-27	65 (15.1)
28-31	4.4)
>31	8 (1.9)
Sex	
Male	191 (44.4)
Female	239 (55.6)
Marital status	
Single	420 (97.7)
Married	10 (2.3)
Tribe	
Efik	102 (23.7)
Ibibio/Annang	88 (20.5)
Igbo	87 (20.2)
Yakurr	13 (3.0)
Others	140 (32.6)
Religion	
Christian	425 (98.8)
Muslim	5 (1.2)
Monthly allowance	
≤50000	333 (77.4)
>50000	97 (22.6)
Smoking status	
Yes	18 (4.0)
No	412 (96)

**Figure 1:**
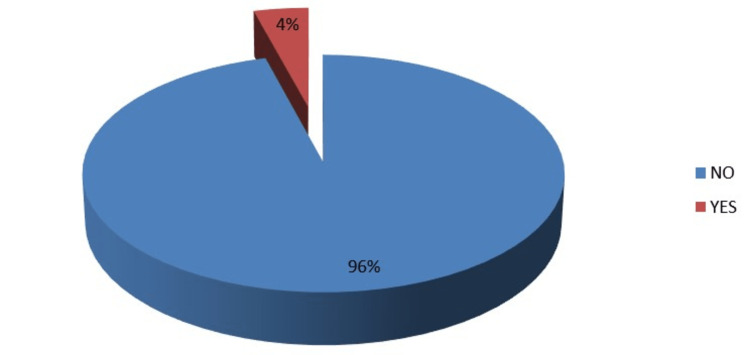
Smoking habits of the respondent

There were more females, 239 (55.6%), than males, 191 (44.4%). Those from the Efik, Ibibio, and Igbo tribes were 102 (23.7%), 88 (20.5%), and 87 (20.2%), respectively, with the rest (32.6%) from other non-indigenous tribes. Most participants, 426 (98.8%), were Christians, and most were single 420 (97.7%). Most of the respondents, 213 (49.5%) were in second year, followed by 103 (24.0%) in third year and 44 (10%) in sixth year. Only 3(0.7%) were in first year. Of the students, 412 (96%), do not smoke, while a few 18 (4.0%) smoked, as seen in Figure 1.

The respondents were categorized into their departments and academic level, as seen in Table 2.

**Table 2:** Respondents’ faculties, departments, and year of study

VARIABLE	FREQUENCY (%)
Faculty	
Medicine	87 (20.2)
Basic medical sciences	85 (19.8)
Arts	87 (20.2)
Social sciences	85 (19.8)
Biological sciences	86 (20.0)
Total	430 (100)
Departments	
Medicine	71 (16.6)
Dentistry	16 (3.6)
Anatomy	42 (9.8)
Biochemistry	43 (10.0)
Botany	43 (10.0)
Microbiology	43 (10.0)
Philosophy	43 (10.0)
Political science	43 (10.0)
English and linguistic studies (ELS)	43 (10.0)
Public administration	43 (10.0)
Total	430 (100)
Year of study	
100	3 (0.7)
200	213 (49.5)
300	103 (24.0)
400	40 (9.3)
500	28 (6.5)
600	44 (10.0)
Total	430 (100)

Knowledge of dental care among the University of Calabar students

Results in Table 3 show respondents’ knowledge of dental care practices. Most of the respondents, 360 (83.7%), knew that the teeth are living parts of the body, while 70 (16.3%) respondents did not know. The majority, 334 (77.7%), knew that the teeth receive blood or oxygen like other body parts, while 96 (22.3%) did not know. A greater percentage, 89.5%, agreed that fresh fruits do not cause disease to the teeth, 6.3% disagreed, and 4.2% did not know if they did or not. Most respondents, 404 (94.0%), agreed that poor dental care could cause disease to the teeth, while 6% did not agree with this. The majority, 391 (91.0%), agree that excessive drinking of water cannot cause disease of the teeth, 18 (4.2%) agreed that it can, while 21 (4.8%) did not know. A higher proportion, 259 (60.2%), have the knowledge that genetic inheritance can cause disease of the teeth, whereas 92 (21.4%) disagreed only 79 (18.4%) did not know. Out of the 430 respondents, 322 (74.9%) knew that trauma to the teeth could cause disease to the teeth, while only 40 (9.3%) are of the opinion that trauma cannot cause disease to the teeth; 68 (15.8%) do not know.

**Table 3:** Knowledge of dental care among respondents

VARIABLE	FREQUENCY (%)
Teeth being a living part of the body.	
Yes	360 (83.7)
No	37 (8.6)
I don’t Know	33 (7.7)
Teeth receiving blood supply like other parts of the body.	
Yes	334(77.7)
No	55 (12.8)
I don’t know.	41 (9.5)
Teeth have diseases like other parts of the body.	
Yes	411 (95.6)
No	14 (3.3)
I don’t know	5 (1.1)
Eating fresh fruits causing disease to the teeth	
Yes	27 (6.3)
No	385 (89.5)
I don’t know	18 (4.2)
Poor dental care causing disease of the teeth.	
Yes	404 (94)
No	20 (4.7)
I don’t know	6 (1.3)
Excess drinking of water cause disease of the teeth	
Yes	18 (4.2)
No	391 (91.0)
I don’t know	21 (4.8)
Genetic inheritance causing disease of the teeth.	
Yes	259 (60.2)
No	92 (21.4)
I don’t know	79 (18.4)
Trauma causing disease to the teeth.	
Yes	322 (74.9)
No	40 (9.3)
I don’t know	68 (15.8)

Attitude towards dental care among the University of Calabar students

Table 4 shows respondents’ attitudes toward dental care (N=430). While the highest proportion of the respondents, 350 (81.4%), strongly agreed that brushing the teeth was necessary, 66 (15.3%) agreed to this, one (0.2%) disagreed, and 13 respondents (3%) strongly disagreed that brushing the teeth was necessary. However, no respondent was indifferent. Most of the respondents, 232 (54%), agreed that visiting the dentist was necessary, while 164 (38.1%) strongly agreed. Very few respondents, eight (1.9%), strongly disagreed that visiting the dentist was necessary, while five (1.2%) disagreed that visiting the dentist was necessary. However, 21 (4.9%) were indifferent. Most respondents, 353 (82%), agreed that bad breath was associated with poor dental care. Of these, 195 (45.3%) respondents agreed strongly, while 158 (36.7%) agreed. A few respondents, 24 (5.6%), were indifferent, 37 (8.6%) disagreed, and 16 (3.7%) disagreed strongly. More than half of the respondents, 241 (56%), agreed strongly that bad breath affects people's self-esteem, while 142 (38%) agreed to this. However, 10 (2.3%) respondents strongly disagreed, 20 (4.7%) disagreed, and 17 (14%) were indifferent. While 83 (19.3%) respondents strongly agreed that brown teeth are a result of poor dental care, 160 (372%) respondents agreed, 83 (19.3%) disagreed, 35 (8.1%) strongly disagreed, and 69 (16%) were indifferent. More than half of the respondents, 324 (75.3%), opined that dentists can help with teeth whitening. Of these, 119 (27.7%) respondents agreed strongly, while 205 (47.7%) just agreed. A small proportion disagreed, of which 44 (10.2%) disagreed while 12 (2.8%) disagreed strongly. A higher proportion of the respondents believed that other diseases of the body could emerge from poor dental care, 61.7%, 20.5% (88) agreed, and 41.2% (177) strongly agreed. 51 (11.9%) disagreed, and 30 (7%) strongly disagreed.

**Table 4:** Attitude towards dental care

Variables	Strongly disagree	Disagree	Indifferent	Agree	Strongly agree	Total
Brushing of the teeth is necessary	13 (3%)	1 (0.2%)	0	66 (15.3%)	350 (81.4%)	430 (100%)
Visiting the dentist is necessary	8 (1.9%)	5 (1.2%)	21 (4.9%)	232 (54%)	164 (38.1%)	430 (100%)
Bad breath is associated with poor dental care	16 (3.7%)	37 (8.6%)	24 (5.6%)	158 (36.7%)	195 (45.3%)	430 (100%)
Bad breath affects people’s self-esteem	10 (2.3%)	20 (4.7%)	17 (4%)	142 (33%)	241 (56%)	430 (100%)
Brown teeth are as a result poor dental care	35 (8.1%)	83 (19.3%)	69 (16%)	160 (37.2%)	83 (19.3%)	430 (100%)
Dentist can help in teeth whitening	12 (2.8%)	44 (10.2%)	50 (11.6%)	205 (47.7%)	119 (27.7%)	430 (100%)
Other diseases of the body can emerge from poor dental care	30 (7%)	84 (19.5%)	51 (11.9%)	177 (41.2%)	88 (20.5)	430 (100%)

Practice of dental care amongst the University of Calabar students

The majority of the respondents, 270 (62.8%), brushed their teeth using the up-down technique, with 75 (17.4 %) brushing left - right and 85 (19.8%) using both techniques (Table 5). Of most respondents, 290 (67.4%) brushed twice a day, 114 (26.5%) brushed once a day, and only 26 (6.1%) brushed after every meal. Approximately half of the students, 217 (50.5%), spent between one and three minutes when brushing their teeth, while 213 (49.5%) spent more time. Replacement of toothbrushes was done every three months by more than half, 248 (57.7%) of the students, with 210 (48.8%) reporting fraying of bristles as the reason for the change. The use of dental floss was found to be quite low, as only 131 (30.5%) reported regular use. Among those who flossed, there were more females, 81 (61.8%), than males. Tongue cleaning was practiced by 413 (96%) respondents, and the toothbrush 392 (91.2%) was the most widely used instrument, followed by tongue cleaner 33 (7.7%). However, a few respondents, five (1.2%), used both toothbrushes and tongue cleaners. Mouth rinsing after a meal was carried out by a large proportion of the respondents, 349 (81.2%), and of these, 351 (91.2%) used water to rinse. Only about 21 (5.5%) used mouthwash.

**Table 5:** Assessment of practice of dental care among the respondents

VARIABLE	FREQUENCY (%)
Tooth brushing technique	
Up-down	270 (62.8)
Left-right.	75 (17.4)
Both	85 (19.8)
Time of brushing teeth	
Morning only	113 (26.3)
Night only	1 (0.2)
Morning and night	290 (67.4)
After every meal	26 (6.1)
Duration of brushing	
1-3 minutes	217 (50.5)
4-5 minutes	175 (40.7)
More than 5 minutes	38 (8.8)
Brushing in front of mirror	
Yes	152 (35.3)
No	278 (64.7)
Frequency of brush change	
Every month	90 (21.0)
Every 3 months	248 (57.7)
Every 6 months	75 (17.5)
More than 6 months	17 (3.8)
Reason for toothbrush change	
Frayed bristles	210 (48.8)
Faded color indicator.	47 (10.9)
No specific reason	173 (40.2)
Use of dental floss? Yes No	131 (30.5) 299 (69.5)
Tongue cleaning?	
Yes	413 (96.0)
No	17 (4.0)
Method of tongue cleaning	
With a tongue cleaner	33 (7.7)
With a toothbrush	392 (91.2)
Both	5 (1.1)
Mouth rinsing after every meal	
Yes	349 (81.2)
No	81 (18.8)
Method of mouth rinsing	
With soft drink	13 (3.0)
With water	351 (81.6)
With mouth wash	21 (4.9)
Others	45 (10.5)

Table 6 shows dental care practice among male and female respondents. Compared with males, most females brush in front of a mirror (64.5% versus 35.5%). This difference was statistically significant (p = 0.006). Although more females compared with males were found to rinse their mouths after meals (55.3% versus 44.7%), there was no statistically significant difference in their proportions (p = 0.81).

**Table 6:** Dental practice among male and female study participants (N=430) *=Statistically significant (p-value = 0.006)

Variable	Male, n (%)	Female, n (%)	Total	Chi-square	P-value	
						Brush before mirror?						
Yes	54 (35.5)	98 (64.5)	152 (100)	7.53	0.006	
No	137 (49.3)	141 (50.7)	278 (100)			
Rinse mouth after meals?						
Yes	156 (44.7)	193 (55.3)	349 (100)	0.06	0.81	
No	35 (43.2)	46 (56.8)	81 (100)			

Table 7 shows a comparison between the higher levels of study (300 and above) and the lower levels of study (200 and below). The lower-level students out of 216 (100%) respondents, 183 (84.7%) had poor practice while 33 (15.3%) had good practice. Among the higher-level student (year three and above), out of 214 (100%) respondents, 146 (68.2%) had poor practice, while 68 (31.8%) had good practice. There was a statistically significant difference between them (chi-square = 16.281, p-value= 0.001).

**Table 7:** Dental practice comparing the year of study (N=430)

Variable	Poor, n (%)	Good, n (%)	Total	Chi-square	P-value	
						Year 1 and 2	183 (84.7)	33 (15.3)	216 (100)	16.281	0.001	
Year 3 and above	146(68.2)	68 (31.8)	214 (100)			

Utilization of dental care services by the University of Calabar students

Table 8 shows the utilization of dental facilities. About half, 218 (50.7%) of the respondents knew at least a dental health facility, while 212 (49.3%) did not know of any dental facility around. Of those who knew, only 118 (54%) had visited a dentist. The majority, however, 312 (72%), had never visited a dental health facility. Most of the visits to the dentist were due to toothache 50 (42%), 20 (17%) were due to tooth extraction, 17(14%) were due to bleeding gum, while 16 (14%) and 15 (13%) were due to medical referral and tooth cleansing, respectively. Of those that benefited from dental services, the majority, 89 (75.4%), were satisfied, while 25 (21.2%) were not.

**Table 8:** Utilization of dental facilities by the students

Variables	Frequency (%)
Knowledge of any dental facility?	
Yes	212 (49.3)
No	218 (50.7)
Ever visited a dentist?	
Yes	118 (27)
No	312 (73)
Reason for visit?	
Toothache	50 (42)
Bleeding gum	17 (14)
Tooth extraction	20 (17)
Tooth cleansing	15 (13)
Medical referral	16 (14)
Satisfaction with the service?	
Yes	89 (75.4)
No	25 (21.2)
Missing	4 (3.4)

**Figure 2:**
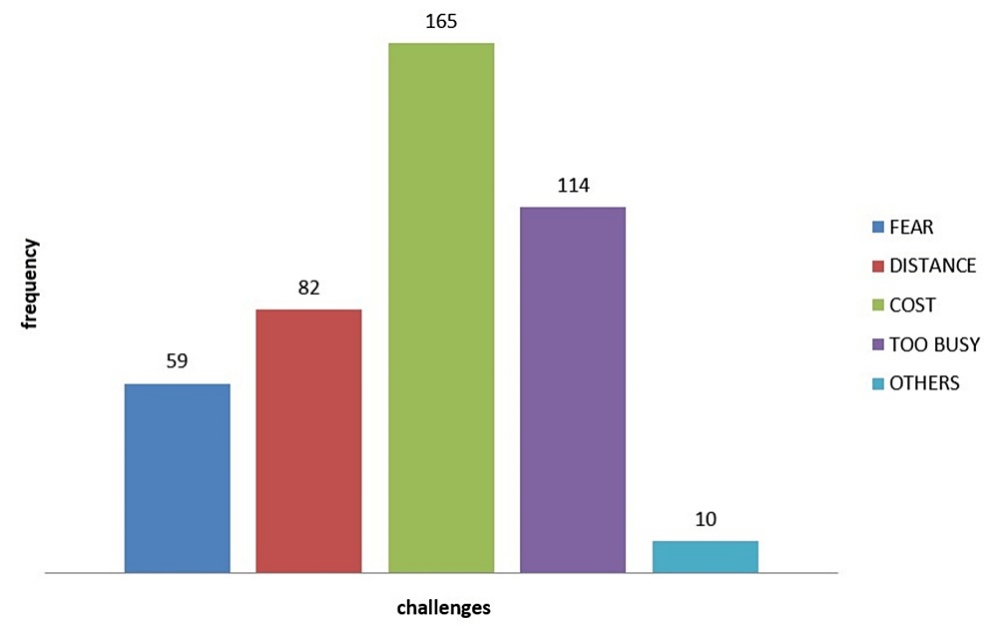
Challenges to utilization of dental service.

Figure 2 shows the challenges in assessing dental services, including fear, distance, cost, busy schedule, and other reasons. A majority, 165 (38.4%) respondents, attributed their challenge to the cost of dental services, followed by those who had busy schedules 114 (26.5%), then distance 82 (19.1%), and fear 59 (13.7%). The last challenge was recorded as those without reason to visit the dentist 10 (2.3%).

**Figure 3:**
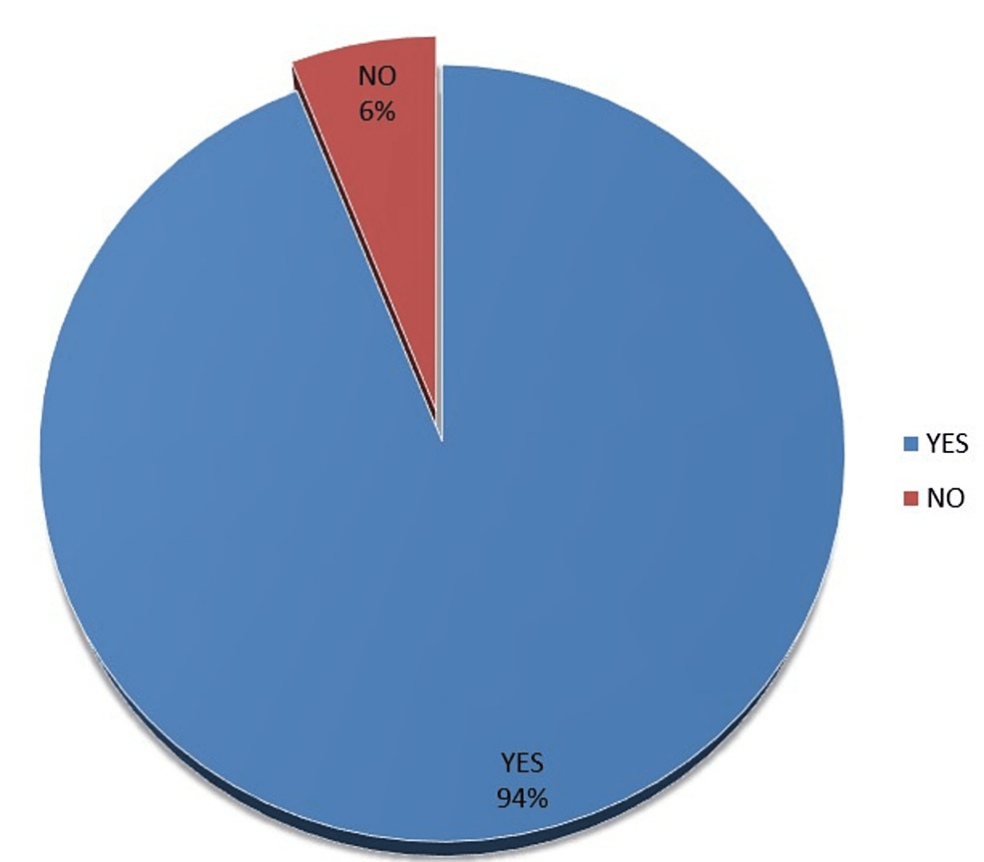
Recommendation of dental services

Figure 3 shows that almost all respondents 404 (94%) would recommend dental service while only 26 (6%) would not.

Binary logistic regression was performed to assess the impact of a number of factors on the practice of dental care of the students at the University of Calabar. The model contained four independent variables (age, year of Study, allowance per semester, and smoking habits) (Table 9). The full model containing all predictors was statistically significant, x^2^ (20.93, N= 430), p < 0.001, indicating that the model could distinguish between respondents who had a good practice and those who had poor practice and correctly classify 76.5% of the respondents.

**Table 9:** Multivariate logistic regression of independent predictors of dental care practice among study participants *=Statistically significant

Variable	Odds Ratio	95% Confidence Interval	P-value
Year of study			
≤2	0.398	0.245-0.646	0.001*
≥3	1.0		
Allowance/Naira			
≤50,000	0.818	0.478-1.400	0.463
>50,000	1.0		
Smoking habit			
Yes	0.313	0.070-1.405	0.129
No	1.0		
Age group/years			
≤25	1.341	0.670-2.684	0.408
>25	1.0		

As shown in Table 9, of all the independent variables, only class level made a uniquely significant contribution to the model. The strongest predictor was age less than 25 recording an odds ratio of 1.34. This implies that those under 25 years are 1.34 times more likely to practice good dental care. This is, however, not statistically significant (95% Confidence interval 0.670-2.684). (The term “good” and “poor” as operationalized in this study by authors' expert judgment. These cut-off points were also influenced by the nature of the questionnaire, the variables being assessed, and the desired interpretation of the overall data collected.)

## Discussion

Knowledge and attitude of dental care

While having knowledge about oral health does not necessarily translate to better health behavior, individuals who possess such knowledge and feel in control of their oral health are more likely to adopt self-care practices [36-49]. In this study, participants demonstrated a high level of knowledge regarding dental care and the consequences of poor dental hygiene, with 94% of respondents acknowledging that poor dental care can lead to dental disease, consistent with findings from a study conducted among dental patients in Saudi Arabia [45]. For instance, with regard to the importance of brushing teeth, 81.5% were in strong agreement that it was essential while 15.3% registered an agreement with the observation. Consequently, 3% of the participants registered a strong disagreement while 1% registered a disagreement. Furthermore, over half of the respondents recognized that poor dental care can cause brown discoloration of teeth, which is in line with results from previous studies [25,46,47]. For instance, of the study participants, 19.3% were in strong agreement that teeth discoloration (brown teeth) was due to poor dental care while 37.2% registered an agreement with the observation. Consequently, 8.1% were in strong disagreement with the view that teeth discoloration was due to poor dental care while 19.3% disagreed with the view, and 16% were indifferent. Thus, the association between poor dental hygiene and discolored teeth was also found to affect self-esteem, a finding supported by previous research. These similarities could be attributed to the fact that the studies were conducted among individuals of similar age groups and shared comparable social influences. Nevertheless, the findings indicate increased awareness and enhanced attitude towards the importance of oral hygiene and dental care among the students.

Interestingly, three-quarters of the respondents agreed that dentists could assist in teeth whitening, which contrasts with a study carried out among undergraduate students at the University of Benin [17], where only about 35.8% of respondents were aware of tooth bleaching, with dentists and the internet as the primary sources of information. The disparity in awareness could be explained by the fact that the Benin study was conducted among part-time students who may have limited exposure to dental services in their immediate environment and spend less time on campus than full-time students in this study.

The practice of dental care

According to the findings of this study on dental care practices, 67.4% of respondents reported brushing twice a day, which is consistent with similar results from a study [20] where 67.2% of participants reported the same. However, the results were slightly lower than those found among dental technology students, who are presumed to have more knowledge than the average non-dental student, with 71.9% of respondents brushing twice a day. This higher level of knowledge could be attributed to privileged academic exposure resulting from their course of study. In contrast, only 40.7% of students in this study brushed for 3-5 minutes, which is lower than the 52.1% reported in [26].

Regarding the use of dental floss, a significantly larger proportion (30.5%) of respondents in this study reported using it compared to studies conducted among university students in Port Harcourt 39 (5.8%) and 37 (3%). Moreover, the study has disclosed that more female students (61.8%) flossed than male students. Although the usage percentage appears more impressive in this study, the frequency of use was not assessed as in the other studies where dental floss was found to be used daily. The lower use of dental floss is indicative of the low awareness level of the importance of dental floss in the maintenance of oral hygiene and dental care, which necessitates the need for awareness creation among students, particularly male students.

Further, regarding tongue cleaning, a significant proportion of the study participants (96%) observed that they practiced tongue cleaning, with 91.2% noting that they utilized a toothbrush as the main tongue-cleaning instrument while 7.7% observed that they used a tongue cleaner. However, 1.2% of the respondents reported using both toothbrushes and tongue cleaners. Based on the findings, one may conclude that tongue cleaning is widely practiced by students and that nearly all students are aware of the importance of tongue cleaning with appropriate tools as an oral hygiene and dental care practice. Similar observation can be made with regard to findings on the importance of mouth rinsing after meals, given that a larger proportion of the participants (81.2%) reported practicing mouth rinsing after every meal, with 91.2% of them using water and 5.5% reporting using mouthwash. Thus, though a larger proportion of the students (81.2%) reported practicing mouth rinsing after every meal, only a smaller proportion of them (5.5%) used mouthwash. The finding indicates the need for increased awareness of the importance of using mouthwash for mouth rinsing after meals.

Furthermore, most respondents (92%) in this study believed that visiting the dentist was necessary, compared to 80% of adolescents in a study conducted in Ibadan [32]. This suggests that with increased age and educational level, the practice of visiting the dentist may improve.

Utilization of dental facility

In this study, only a small proportion of respondents (27%) reported visiting a dentist, which is significantly lower than the 58% reported among students in India [35]. Out of the 116 respondents who reported visiting a dentist, only 15 (13%) did so for routine dental check-ups, which is lower than the 18% reported among health workers in Benin City [41]. In the present study, slightly above half (50.7%) of the participants reported knowing a dental facility, even as 49.3% reported not knowing any dental facility in the area. However, of the participants who knew where the facilities were, only 54% reported visiting a dentist. The above findings indicate a lower uptake of dentist services by students, given the low awareness of the existing dental facilities in the region and the low visit rates to dentists. Nonetheless, it should be noted, however, that the health workers' higher rate of visits may be due to their better health-seeking attitude, as the study was carried out in a health facility.

Of those who did visit the dentist in this study, the majority (42%) did so because of toothache or pain, which is consistent with previous studies [35,36]. The main reason for poor utilization of dental services was cost (38.4%), followed by lack of time (26.5%), which is similar to findings in a study among the University of Lagos students. In that study, the main reason for poor utilization was a lack of perceived need for treatment (53%), but lack of time was also a significant barrier. Fear of anticipated discomfort was also a common barrier, consistent with the University of Lagos study. Interestingly, most respondents in this study would recommend the use of dental facilities, which is consistent with the University of Lagos study. Additionally, those under 25 years old were 1.34 times more likely to practice good dental care, including visiting a dentist, although this finding was not statistically significant. This contrasts with findings among adults in Benin City, where 25 to 34-year-olds were twice as likely as those aged 18-24 years to have visited a dentist after adjusting for sex and employment. One possible explanation for this discrepancy is that younger students in this study may have more time and more parental influence on health-seeking behavior than older students.

Study limitation

Some limitations that may have influenced the study's outcome are self-report bias during questionnaire data collection. Self-report data can be subject to biases, such as social desirability bias or recall bias, which may impact result validity. Additionally, the questionnaires used in the study were validated through content validation and construct validation, given that construct validation is likely to result in poor construct operationalization even as content validation might fail to be exhaustive and selective. Participants may misinterpret questions or provide incomplete or inaccurate responses. Furthermore, questionnaires are primarily used to gather data on associations and relationships between variables but do not establish causal relationships due to the involvement of other factors. Lastly, the study's conclusion may have been affected by the limitation of not conducting multivariate analysis, including odds ratios and 95% confidence intervals, for certain variables analyzed in the regression model. Although the study did not aim to determine causality but rather knowledge, the omission of multivariate analysis could impact the overall statements in the study's conclusion. Nevertheless, the study's outcome measures were considered sufficient for achieving the study's objective. Moreover, the lack of the perceived need for dentist visits can be considered a major limitation of the present study, given that it made it increasingly difficult to assess the student's perception of dental care services and practices offered by dentists.

Recommendation

According to the findings of this study, we recommend that university authorities take proactive measures to promote dental health among students. Specifically, universities should consider incorporating information on dental health into their student handbooks and offering dental care education to first-year students. Additionally, dental facilities should be established within school medical centers, and dental services should be subsidized for university students. To further encourage dental health, students should be given priority when they visit the dental facility.

Furthermore, we suggest that the government sponsor more jingles on radio and television to promote good dental health. Prospective studies should focus on how World Dental Day, which is marked on March 20, aids in improving attitudes and practices related to oral hygiene and dental care. By implementing these recommendations, we can help ensure that students receive the support and resources they need to maintain good dental health while pursuing their academic goals.

## Conclusions

The practice of dental care and utilization of dental care services among the University of Calabar students was low in spite of adequate knowledge. The use of dental floss and mouthwash was uncommon among the students and only a little over a quarter had ever visited the dentist. The most common reason for not visiting the dentist regularly was perceived as high cost. Another factor found to be a common barrier to the utilization of dental services and visits to the dentist was a lack of time. Considering the impact of regular visits to the dentist on the prevention of dental diseases and preservation of oral health, it is therefore pertinent that these barriers be addressed both at the University level and in society at large.
